# An Abnormally High Neutrophil-to-Lymphocyte Ratio Is Not an Independent Outcome Predictor in AQP4-IgG-Positive NMOSD

**DOI:** 10.3389/fimmu.2021.628024

**Published:** 2021-02-23

**Authors:** Edgar Carnero Contentti, Guillermo Delgado-García, Juan Criniti, Pablo A. López, Juan Pablo Pettinicchi, Edgardo Cristiano, Jimena Miguez, Edgar Patricio Correa-Díaz, Marcelo Oswaldo Álvarez Pucha, Joselyn Elizabeth Miño Zambrano, Enrique Gómez-Figueroa, Verónica Rivas-Alonso, José Flores-Rivera, Verónica Tkachuk, Alejandro Caride, Juan Ignacio Rojas

**Affiliations:** ^1^Neuroimmunology Unit, Department of Neuroscience, Hospital Alemán, Buenos Aires, Argentina; ^2^Instituto Nacional de Neurología y Neurocirugía, Mexico City, Mexico; ^3^Centro de Esclerosis Múltiple de Buenos Aires (CEMBA), Buenos Aires, Argentina; ^4^Universidad Central del Ecuador, Quito, Ecuador; ^5^Pontificia Universidad Católica del Ecuador, Quito, Ecuador; ^6^Hospital Carlos Andrade Marín, Quito, Ecuador; ^7^Neuroimmunology Unit, Department of Neurology, Hospital de Clínicas “José de San Martín”, Buenos Aires, Argentina

**Keywords:** neuromyelitis optica spectrum disorder, predictors, expanded disability status scale, relapses, Latin America, brain MRI, biomarkers

## Abstract

**Background:**

The neutrophil-to-lymphocyte ratio (NLR) has been investigated in many autoimmune conditions as a biomarker of inflammation and/or disease activity. The role of NLR in AQP4-IgG-positive neuromyelitis optica spectrum disorders (NMOSD) is far from clear. In this study, NLR was evaluated in patients with AQP4-IgG-positive NMOSD at disease onset and its prognostic impact was subsequently assessed.

**Methods:**

In this multicenter study, we retrospectively included all recent/newly diagnosed treatment-naïve patients with AQP4-IgG-positive NMOSD (n=90) from three different countries in Latin America (LATAM): Argentina, Ecuador, and Mexico. NLR was compared between AQP4-IgG-positive NMOSD and healthy controls (HC, n = 365). Demographic, clinical, paraclinical (including imaging), and prognostic data at 12 and 24 months were also evaluated. Multivariate regression analysis was used to describe and identify independent associations between the log-transformed NLR and clinical (relapses and EDSS) and imaging (new/enlarging and/or contrast-enhancing MRI lesions) outcomes.

**Results:**

NLR was higher in NMOSD patients during the first attack compared with HC (2.9 ± 1.6 vs 1.8 ± 0.6; p<0.0001). Regardless of immunosuppressant’s initiation at disease onset, NLR remained higher in NMOSD patients at 12 (2.8 ± 1.3; p<0.0001) and 24 (3.1 ± 1.6; p<0.0001) months. No association was found at 12 and 24 months between the log-transformed NLR and the presence of relapses, new/enlarging and/or contrast-enhancing MRI lesions, and/or physical disability.

**Conclusions:**

In this cohort of LATAM patients with AQP4-IgG-positive NMOSD, NLR was abnormally high in attacks but also during follow-up. However, a high NLR was not an independent predictor of clinical or imaging outcomes in our models.

## Introduction

Neuromyelitis optica spectrum disorder (NMOSD) is a rare and disabling condition characterized by inflammatory attacks, especially involving the optic nerves and spinal cord (1). NMOSD is mainly regarded as a disorder of the humoral immunity, as it is driven by autoantibodies (1, 2). The classic example of this abnormal humoral response is the presence of the autoantibody against aquaporin-4 (AQP4-ab), an abundant water channel mainly expressed in astrocytic foot processes.

White blood cells (WBC) and their subtypes has been proposed as biomarkers for inflammation and/or disease activity. Neutrophil-to-lymphocyte ratio (NLR) represents a combination of two of these markers, and is superior to other WBC-derived parameters, due to its stability (3, 4). NLR has been studied in many autoimmune disorders such as systemic lupus erythematosus (SLE) (5), Sjögren syndrome (SS) (6), Behçet disease (7), Hashimoto disease (8), multiple sclerosis (MS) (9–12), among others. Co-existing autoimmune disorders, including both SLE and SS, are not uncommon in AQP4-ab-positive NMOSD (13). In addition, degranulating perivascular neutrophils have been described in NMOSD lesions, suggesting a central role of neutrophils in the pathogenesis of early NMOSD lesions (14). Compared to MS, neutrophils are increased in the CSF of patients with NMOSD during relapses (15). These two findings may be helpful in differentiating between NMOSD and other inflammatory conditions, especially MS. In a mouse model of AQP4-ab-positive NMOSD, reduced neuroinflammation and AQP4 loss was reported in neutropenic mice and, on the contrary, granulocyte colony stimulating factor increased the severity of NMOSD lesions (14). These lesions also decreased after the administration of neutrophil protease inhibitors (14). Additionally, altered functionality of neutrophils in NMOSD patients was also reported (15). However, the role of NLR in AQP4-ab-positive NMOSD is currently far from clear and, to the best of our knowledge, only two cross-sectional studies have specifically addressed this issue (16, 17).

NLR is an easily obtained parameter that could act as a biomarker of inflammation and/or disease activity in NMOSD. Therefore, the aim of this study was to evaluate NLR in a Latin American (LATAM) cohort of patients with AQP4-ab-positive NMOSD at disease onset and then to assess the prognostic role of this potential biomarker at two years.

## Methods

In this multicenter study, we retrospectively included patients with a first NMOSD attack who fulfilled the 2015 diagnostic criteria (1) in order to evaluate NLR and its potential role as an independent predictor for relevant outcomes. Patients from Argentina (n=42), Ecuador (n=30), and Mexico (n=18) were included. As the NLR is influenced by different physiological and medical conditions, as well as medications, the following patients were deemed ineligible to participate: subjects younger than 18 years of age or older than 80 years, those pregnant, with evidence of infection, other autoimmune (including rheumatoid arthritis, SS, SLE, and inflammatory bowel disease), cardiometabolic (including diabetes mellitus, hypertension, and dyslipidemia) or liver diseases, malignancies, hematologic conditions, or blood transfusions during the last four months, as well as users of antiplatelet medications (such as aspirin and clopidogrel). We only included patients with a first-ever NMOSD attack and, therefore, only treatment-naïve patients. In addition, all blood samples were obtained before the first dose of steroids. Only patients with complete work-ups were included.

Data collection included demographics and core clinical characteristics at onset. Disability and new/enlarging T2 and/or gadolinium-enhancing (Gd+) lesions on MRI were assessed at onset, 12 and 24 months. The frequency of clinical relapses was evaluated at 12 and 24 months. In LATAM reference centers, it is a standard practice to perform annual MRIs as a complementary evaluation during follow-up (18). Thus, MRIs performed approximately at 12 and 24 months were compared with the baseline MRI performed during the first attack. In addition, clinical follow-up (usually including general lab tests) is systematically performed every 3–6 months in LATAM. Demographic data included age, gender, and ethnicity. Ethnicity was used as a dichotomous variable and patients were classified as either Caucasian or non-Caucasian, as previously described (19). Non-Caucasian subgroups included Mestizo (mixed Caucasian and Aboriginal ancestry), Afro descendant (including mixed Caucasian and African ancestry), Aboriginal (indigenous peoples of the Americas), and Asian (including also mixed Caucasian and Asian ancestry). A relapse was defined as an acute event of neurologic symptoms lasting 24 h or more, presenting at least 30 days after the previous attack. NMOSD core clinical characteristics at onset were defined as follows: acute transverse myelitis (ATM), optic neuritis (ON), area postrema syndrome (APS), brainstem syndrome (BSS), narcolepsy or diencephalic syndrome (DS), and cerebral syndrome (CS). Phenotypic combinations at onset were also documented. Clinical course at follow-up was defined as monophasic (e.g., isolated clinical event) or polyphasic (i.e., recurrent). Disability was estimated using the Expanded Disability Status Scale (EDSS) (20).

Blood samples were obtained and recorded within 24 h of admission at disease onset. Data containing laboratory results such as complete blood count (CBC) were included. NLR at baseline, 12 months and 24 months was evaluated in both patients with NMOSD and healthy controls (HC). After the first attack, all patients were started on immunosuppressant treatment (IST). For those started on azathioprine (AZA) or mycophenolate mofetil (MMF), a concomitant oral steroid was used for 3–6 months while steroid-sparing therapy reached full efficacy. As they were on IST, follow-up routine lab tests were requested. These tests (including a CBC) were reviewed approximately at 12 and 24 months after the first attack. HC [median age 40 (range: 18–65) years] were recruited in Argentina (2012–2017) among healthy subjects who underwent a routine physical examination and general blood tests for their annual physicals. This is part of a preventive healthcare program in Argentina. These medical records were retrieved in an anonymized manner using only filters for age and reason for visit (annual physical). Therefore, gender is unknown. NLR was calculated as the absolute count of neutrophils divided by the absolute count of lymphocytes from peripheral blood samples before any treatment has been started. Serum AQP4-ab was tested by cell-based assay and only patients with positive results were included.

NMOSD typical lesions on brain MRI were classified as follows (21, 22): optic nerve lesions (extending over 50% of the optic nerve length or presenting bilaterally increased T2 signal or involving optic chiasm), brainstem/cerebellum lesions (periependymal surfaces of the fourth ventricle and cerebellar peduncle), area postrema lesions (dorsal medulla or contiguous with an upper cervical spinal cord lesion), diencephalic lesions (hypothalamus and/or thalamus or periependymal surfaces of the third ventricle), periependymal lesions surrounding the lateral ventricles (at least 50% of the length of the corpus callosum), and corticospinal tract lesions hemispheric white matter lesions (> 3 cm in largest diameter). Spinal cord MRI abnormalities were classified as follows: longitudinally extensive transverse myelitis (LETM, lesions ≥ 3 spinal vertebral bodies), short-segment transverse myelitis (SSTM, only one lesion < 3 vertebral bodies), and multisegmental (MSL, two or more lesions ≤ 2 and/or ≥ 3 noncontiguous vertebral bodies). In addition, patients with MRI lesions were classified as Gd+ or gadolinium negative, according to the contrast enhancement.

### Statistical Analyses

Statistical analysis was performed using STATA 13.0 (StataCorp, TX). Categorical variables are reported as absolute count/frequency (percentage), while continuous variables are summarized as mean ± standard deviation (SD) or median (IQR), according to their distribution. Distribution was assessed using a combination of the following: visual inspection of histogram and normal probability plot, median, mean, skewness, kurtosis and the Shapiro-Wilk test. NRL was natural log (base e) transformed to fit the normal distribution.

Neutrophil and lymphocyte counts, as well as NLR, were compared using the t-test or Mann-Whitney U test, as appropriate. Regression analysis was used to describe and identify independent associations between log-transformed NLR and clinical (relapses and EDSS change) as well as MRI activity (new/enlarging and/or contrast-enhancing MRI lesions). To account for multiple observations on each subject, we used a lineal mixed-effects model with a subject specific random intercept. First, we analyzed the association between NLR and the described outcomes, and then we evaluated separate models with different adjustments (including a combination of age, gender, country of origin, ethnicity, clinical course, and maintenance treatment). We further explored the association of NLR and EDSS assuming a Poisson distribution and dichotomizing it. For all the analyses, significance level (p) was set at less than 0.05.

## Results

Ninety AQP4-ab-positive patients and 365 HC were included. Twelve AQP4-ab-positive NMOSD patients were excluded due to diabetes mellitus (n = 3), arterial hypertension (n = 2), and concomitant autoimmune conditions (n = 7). General characteristics of this LATAM cohort are summarized in **Table 1**.

**Table 1:** Baseline characteristics of LATAM patients with AQP4-IgG-positive NMOSD.

	NMOSD (n = 90)
**Age at disease onset, years (mean, SD)**	42.3 ± 14.3
**Female (n, %)**	75 (83.3%)
**Ethnicity (n, %)** Caucasian Non-Caucasian Mestizo Afrodescendant	14 (15.5%) 76 (84.4%) 75 (83.3%) 1 (1.1%)
**Country of origin (n, %)** Argentina Ecuador Mexico	42 (46.6%) 30 (33.3%) 18 (20%)
**Clinical phenotype at onset (n, %)** ON TM ON + TM Other combinations	40 (44.4%) 24 (26.6%) 15 (16.6%) 11 (12.2%)
**Time to first relapse, months (mean ± SD | median [IQR])**	13.0 ± 14.0 | 9 (2–9)
**Relapses after first attack, n (mean ± SD | median, [IQR])**	2.6 ± 1.5 | 2 (1–4)
**Relapses (n, %)** At 1 year At 2 years	37 (41.1%) 26 (30.9%)
**Disability (median [IQR])** EDSS at presentation EDSS at 1 year EDSS at 2 years	3.5 (2–5) 3.5 (2–5) 3.5 (2–5)
**Brain MRI at presentation (n, %)** Typical brain MRI lesion	54 (60%)
**Spinal cord MRI at presentation (n, %)** LETM STM	26 (28.9%) 8 (8.9%)
**Acute treatment (n, %)** Plasma exchange (PLEX) Corticosteroids + PLEX	1 (1.1%) 24 (26.6%)
**Initial maintenance therapy (n, %)** Azathioprine Mycophenolate Rituximab	23 (25.5%) 9 (10%) 58 (64.5%)

ON, optic neuritis; TM, transverse myelitis; EDSS, Expanded Disability Status Scale; MRI, magnetic resonance imaging; LETM, longitudinally extensive TM; STM, short-segment TM.

**Figure 1:**
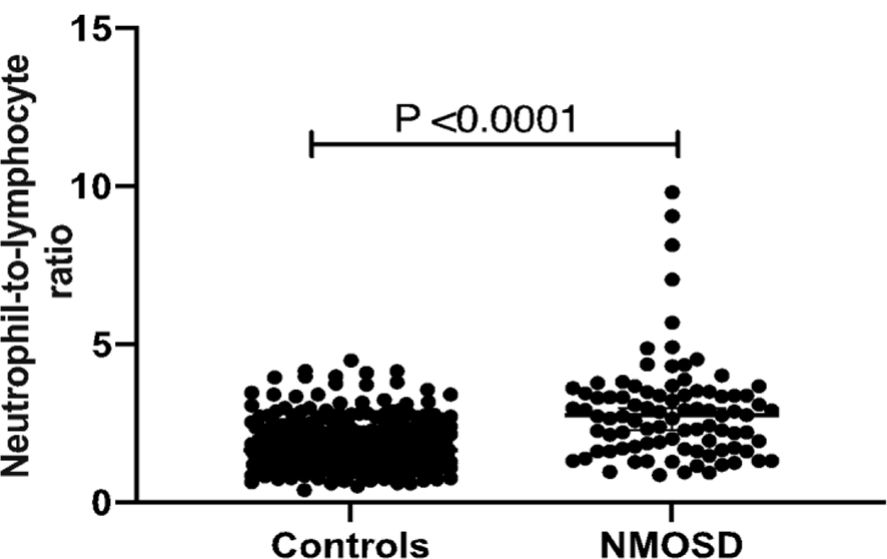
Baseline NLR in HC and NMOSD patients. In patients, this NLR was taken during the first attack. The results of a two-tailed t-test are shown.

NLR was higher in NMOSD patients during the first attack compared to HC (2.9 ± 1.6 vs. 1.8 ± 0.6; p<0.0001) (**Figure 1**). Regardless of immunosuppressants’ initiation at disease onset, NLR continued to be higher in NMOSD patients at 1 (2.8 ± 1.3; p<0.0001) and 2 (3.1 ± 1.6; p<0.0001) years (**Table 2**).

**Table 2:** Complete blood count (CBC)-derived parameters, including NLR.

	NMOSD	HC	P-value
**ANC at onset** **ALC at onset** **ANC at 1 year** **ALC at 1 year** **ANC at 2 years** **ALC at 2 years**	4,406.1 ± 1,716.1 1,702.5 ± 684.3 4,076.8 ± 1,257 1,457.7 ± 880.3 4,681.3 ± 1,530 1,510.6 ± 915.6	3,809.8 ± 1,272.8 2,226.4 ± 651.7	0.01 <0.0001
**NLR at onset** **NLR at 1 year** **NLR at 2 years**	2.9 ± 1.6 2.8 ± 1.4 3.1 ± 1.6	1.8 ± 0.7 – –	<0.0001

ALC, Absolute lymphocyte count; ANC, Absolute neutrophil count; HC, Healthy controls; NLR, Neutrophil to lymphocyte ratio; NMOSD, Neuromyelitis optica spectrum disorder.

No association was found at 1 and 2 years between the log-transformed NLR and relapses, new/enlarging and/or contrast-enhancing MRI lesions, or physical disability when considering multiple observations (**Table 3**). In addition, these results were further adjusted for combinations of age, gender, country of origin, ethnicity, clinical course, and initial maintenance therapy (IMT), and these findings were similar to those presented in **Table 3** (data not shown). Specifically, IMT (rituximab *versus* others) was not identified as an effect modifier of the relationship between the log-transformed NLR and NMOSD outcomes in the mixed-effects regression model.

**Table 3:** Log-transformed neutrophil to lymphocyte ratio as univariate predictor of NMOSD outcomes.

	OR	95% CI	p
Relapses at 1 year	0.66	0.28–1.58	0.362
Relapses at 2 years	0.76	0.30–1.93	0.569
New/enlarging and/or contrast enhancing lesion(s) on MRI at 1 year	1.72	0.58–5.04	0.321
New/enlarging and/or contrast enhancing lesion(s) on MRI at 2 years	0.42	0.13–1.29	0.130
Dichotomized EDSS*	1.09	0.47–2.52	0.829
EDSS at 1 year (lineal)	−0.21	−1.04–0.61	0.604
EDSS at 1year (Poisson)	−0.06	−0.28–0.16	0.595
EDSS at 2 years (lineal)	−0.15	−1.01–0.69	0.717
EDSS at 2 years (Poisson)	−0.04	−0.44–0.18	0.702

EDSS, Expanded Disability Status Scale; MRI, magnetic resonance imaging. *EDSS was also dichotomized into Lower (<4) and higher (≥4).

## Discussion

In this study, compared to HC, NLR is higher in LATAM patients with AQP4-ab-positive NMOSD at disease onset. In general, this finding is similar to that previously reported by Lin et al. in a cohort of Chinese adult patients (17). However, we only included NMOSD patients during their first attack and, therefore, all of them were treatment-naïve at disease onset. This stringent selection criterion was specifically chosen to avoid the potential cofounding effect of chronic immunosuppression. RTX-induced neutropenia has been reported occurring usually several weeks following the administration of this monoclonal antibody. Although the pathophysiology remains unclear, this side effect appears to be transient and self-limited (23). Cytopenias induced by AZA and MMF are also well-known. By contrast, steroids may also have an artefactual effect on NLR (16), due to a spurious increase in neutrophil count, but in the present study all blood samples were obtained before the first dose of steroids. In order to further support this approach, follow-up NLRs at 1 and 2 years were also collected, and they persisted elevated even two years after the first attack and under maintenance therapy. Likewise, in this multicenter cohort, no evidence was found to support the role of IMT as a predictor of NMOSD outcomes.

Benetou et al. recently reported a study on NLR in pediatric demyelinating diseases and included six patients with AQP4-ab-positive NMOSD (16). Compared to HC, these patients had a higher NLR not only during attacks, but also in remission. The latter observation is in line with our findings, since NLR was still elevated one and two years after the first attack in our cohort of adult patients. An additional study addressed NLR in optic neuritis (9). However, serostatus was not reported. In this multicenter LATAM cohort, no evidence was found to support the role of initial clinical phenotypes as predictors of NMOSD outcomes.

Neutrophil infiltration plays a relevant role in the pathogenesis of NMOSD (14, 24), particularly during NMOSD relapses (15) and, in our study, one way to interpret this high NLR is as a relative increase in circulating neutrophils (**Table 2**). In an *in vitro* study of AQP4-ab-positive NMOSD, bystander cytotoxicity has been seen with neutrophil-mediated antibody-dependent cellular cytotoxicity (25). In addition, neutrophil‐related chemokines are elevated in patients with NMOSD during relapses (24). Around 40% of patients in this cohort experienced relapses on maintenance therapy. This could explain, at least partially, the increased NLR seen during follow-up. Interestingly, there was no change in the EDSS at follow-up. Different approaches were used to address this issue (e.g., dichotomization, Poisson, and normal-like distribution assumption) and no statistically significant differences were found. Another explanation is the limitations inherent to the EDSS. It is strongly focused on ambulation and does not sufficiently reflect visual impairment. Therefore, patients with isolated ON cannot reach an EDSS score higher than 4, even if complete bilateral visual loss is present (26).

At that point our analysis seems to indicate that, in AQP4-ab-positive NMOSD, NLR might be a potential surrogate for inflammation and not, strictly speaking, for disease activity (**Table 2**), contrary to what has been described in previous studies (16, 17). As this matter was not entirely clear, our study went one step further and directly assessed the prognostic impact of an abnormally high NRL in LATAM patients with AQP4-ab-positive NMOSD.

Therefore, this key point was evaluated by different dedicated multivariate models, adjusting the results for different variables, including gender, ethnicity, country of origin, disease duration, clinical course, and maintenance therapy. At the end, it was not possible to demonstrate that NLR was an independent predictor for worse clinical or neuroimaging outcomes at one or two years. This is different to what was previously described for other neurological conditions, e.g., MS (9–12), which is not completely unexpected since the pathophysiology behind these two conditions is clearly different (27). For instance, neutrophils have an activated phenotype in both NMOSD and MS. However, in patients with NMOSD, these cells also show reduced adhesion and migratory capacity as well as decreased production of reactive oxygen species and degranulation (15).

Besides its retrospective nature, restricting our recruitment only to AQP4-ab-positive patients represents the main limitation of this study. Factors accounting for this restriction have already been discussed by our team and also by others (27–30). On the other hand, this limitation is also an advantage, as it secured the homogeneity of our cohort. A short follow up period (i.e., 2 years) may also be regarded as an additional limitation. However, enough disease activity was captured during this period and, therefore, it was possible to conduct the intended analyses. Finally, our follow-up blood samples at 1 and 2 years were drawn regardless of the clinical status (i.e., remission or relapse). Blood samples drawn specifically during remission and relapses might provide more accurate estimates and should be considered in the design of future studies.

In summary, this is the largest study to date specifically focused on this topic and confirmed that NLR is abnormally high in adult patients with AQP4-ab-positive NMOSD during both initial attack and subsequent years. However, in our LATAM cohort, NLR does not appear to act as an independent predictor of worse outcomes. These findings may shed some light on the potential pathogenesis of NMOSD and suggest that NLR may be quite limited as a biomarker of disease activity. Further studies including different ethnicities and geographical origins are needed to assess the generalizability of our conclusions.

## Data Availability Statement

The raw data supporting the conclusions of this article will be made available by the authors, without undue reservation.

## Ethics Statement

Ethics committee approval was obtained for each participating center and a written informed consent (according to each committee, if necessary) was obtained from all participants before data collection.

## Co-Investigators/Affiliated Co-Authors

†Affiliated members of The Guthy-Jackson Charitable Foundation International Clinical Consortium (GJCF-ICC) who revised the manuscript for intellectual content: **Hesham Abboud**, MD, PhD, University Hospitals of Cleveland, Case Western Reserve University, Cleveland, OH, USA; **Raed Alroughani**, MD, FRCPC, FAAN, Amiri Hospital Kuwait City, Kuwait; **Metha Apiwattanakul**, MD, Prasat Neurological Institute, Bangkok, Thailand; **Jeffrey Cohen**, MD, Cleveland Clinic, Cleveland, OH, USA; **Joachim Havla**, MD, LMU Hospital, Munich, Germany; **Jyh Yung Hor**, MD, Penang General Hospital, Penang, Malaysia; **Raffaele Iorio**, MD, PhD, Fondazione Policlinico Universitario A. Gemelli IRCCS, Rome, Italy; **Anu Jacob**, MD, Cleveland Clinic Abudhabhi, Abu Dhabi, United Arab Emirates; **Najib Kissani**, MD, Marrakech Medical School, Cadi Ayyad University, Marrakech, Morocco; **Michael Levy**, MD, PhD, Massachusetts General Hospital, Boston, MA, USA; **Sara Mariotto**, MD, PhD, University of Verona, Italy; **Marcelo Matiello**, MD, MSc, Harvard Medical School, Boston, MA, USA; **Esther Melamed**, MD, PhD, Dell Medical School, UT Austin, Austin, TX, USA; **Veronika E. Neubrand**, PhD, University of Granada, Granada, Spain; **Celia Oreja-Guevara**, MD, PhD, Hospital Clinico San Carlos, Madrid, Spain; **Friedemann Paul**, MD, Charité University, Berlin, Germany; **Anne-Katrin Pröbstel**, MD, PhD, University Hospital Basel, Switzerland; **Peiqing Qian**, MD, Swedish Medical Center, Seattle, WA, USA; **Sasitorn Siritho**, MD, Siriraj Hospital, Mahidol University, Bangkok, Thailand; **Terry J. Smith**, MD, University of Michigan Medical School, Ann Arbor, MI, USA; **Pablo Villoslada**, MD, Stanford University School of Medicine, Stanford, CA, USA; **Dean Wingerchuk**, MD, Mayo Clinic, Scottsdale, AZ, USA; **Michael R. Yeaman**, PhD, 1) Los Angeles Biomedical Research Institute at Harbor-University of California at Los Angeles (UCLA) Medical Center, Torrance, CA, USA, 2) David Geffen School of Medicine at UCLA, Los Angeles, CA, USA.

## Author Contributions

ECC and JR: designed/conceptualized the study, analyzed the data, interpreted the data, and drafted and revised the manuscript for intellectual content. GD-G: collected and interpreted the data and drafted and revised the manuscript for intellectual content. JC: collected and interpreted the data, analyzed the data, and revised the manuscript for intellectual content. PL, JP, EC, JM, EC-D, MÁ, JMZ, EG-F, VR-A, JF-R, VT, and AC: collected and interpreted the data, and revised the manuscript for intellectual content. All authors contributed to the article and approved the submitted version.

## Funding

The Article Processing Charge was funded by The Guthy-Jackson Charitable Foundation.

## Conflict of Interest

ECC has received reimbursement for developing educational presentations, educational and research grants, consultation fees, and/or travel stipends from Biogen, Bayer, Genzyme, Merck, Novartis, Roche, Raffo, and Teva. GD-G has received research grants/salary support from the Consejo Nacional de Ciencia y Tecnología (Mexico), Universidad Nacional Autónoma de México, and Fundación Carlos Slim. JC serves as a Health outcomes manager for GlaxoSmithKline. PL has received reimbursement for developing educational presentations, educational and research grants, consultation fees, and/or travel stipends from Biogen, Bayer, Genzyme, Merck, Novartis, Roche, Raffo, and Teva. JP has received reimbursement for developing educational presentations, educational and research grants, consultation fees, and/or travel stipends from Biogen, Bayer, Genzyme, Merck, Novartis, Roche, Raffo, and Teva. EC has received reimbursement for developing educational presentations, educational and research grants, consultation fees, and/or travel stipends from Biogen, Bayer, Genzyme, Merck, Novartis, Roche, and Teva. JM has received reimbursement for developing educational presentations, educational and research grants, consultation fees, and/or travel stipends from Bayer, Merck, and Novartis. VR-A has received reimbursement for developing educational presentations, educational and research grants, consultation fees,and/or travel stipends from Roche, Sanofi, Merck, Stendhal, Biogen, Novartis, and Allergan. JF-R has received reimbursement for developing educational presentations, educational and research grants, consultation fees, and/or travel stipends from Roche, Sanofi, Merck, Stendhal, Biogen, Novartis, Terumo BCT, Bayer, and Teva. VT has received reimbursement for developing educational presentations, educational and research grants, consultation fees, and/or travel stipends from Biogen, Bayer, Genzyme, Merck, Novartis, Roche, Raffo, and Teva. AC has received reimbursement for developing educational presentations, educational and research grants, consultation fees, and/or travel stipends from Biogen, Bayer, Genzyme, Merck, Novartis, Roche, Raffo, and Teva. JR has received reimbursement for developing educational presentations, educational and research grants, consultation fees, and/or travel stipends from Biogen, Bayer, Genzyme, Merck, Novartis, Roche, and Teva.

The remaining authors declare that the research was conducted in the absence of any commercial or financial relationships that could be construed as a potential conflict of interest.
